# Safe sex self-efficacy and safe sex practice in a Southern United States College

**DOI:** 10.15171/hpp.2017.14

**Published:** 2017-03-05

**Authors:** Ovuokerie Addoh, Eveleen Sng, Paul D. Loprinzi

**Affiliations:** ^1^Exercise Psychology Laboratory, Department of Health, Exercise Science and Recreation Management, The University of Mississippi, University, MS 38677, USA; ^2^Department of Health, Exercise Science and Recreation Management, Physical Activity Epidemiology Laboratory, Exercise Psychology Laboratory, The University of Mississippi, University, MS, USA

**Keywords:** Condom use, HIV, Prevention, Sexual health, Southern college

## Abstract

**Background:** The purpose of this study was to assess the association between safe sex self-efficacy and safe-sex practice in a Southern college setting.

**Methods:** Multivariable logistic regression models were used to examine the association between safe sex self-efficacy in four domains (mechanics, partner disapproval, assertiveness, intoxicants) and safe sex practice (outcome variable).

**Results:** For every 1-unit increase in the composite condom use self-efficacy score, there was an 8% increase in the odds of being beyond the median safe-sex practice score (odds ration [OR]: 1.08, 95% CI: 1.02-1.15). Additionally, for every 1-unit increase in intoxicants self-efficacy score, there was a 31% increase in the odds of being beyond the median safe-sex practice score (OR: 1.31, 95% CI: 1.08-1.58).

**Conclusion:** A greater degree of safe-sex self-efficacy is associated with increased odds of safe-sex practice. These findings are informative for the development of targeted approaches to foster safe-sex behavior in Southern US colleges.

## Introduction

Safe sex practices refer to sexual activity and especially sexual intercourse in which various measures such as the use of latex condoms or the practice of monogamy, are taken to avoid sexually transmitted infections (STIs), such as the human immunodeficiency virus (HIV).^1^ Promotion of safe sex practices among college students is particularly important considering the burden of HIV and other STIs in this population with reportedly 26% of new cases of HIV in the United States seen among US youth^2^ and nearly half of the 20 million new STIs each year seen in this demographic as well.^3^ In addition to age distribution, STI incidence and prevalence varies based on other sociodemographic variables such as the geographical location – about the highest rates of STIs in the United States is seen in Southern part of the country.^4-6^

The college years are particularly critical considering the interplay of neo-independence (autonomy), exuberance, exploration with alcohol, drugs and sexual intercourse.^7^ In order to efficiently promote safe sex practices particularly among college students, we must understand the determinants of this behavior. Various behavior models have shown some utility in predicting safe-sex practices among college students, including the social cognitive model,^8^ an integrated model^9^ using constructs from the aforementioned model, the health belief model, theories of reasoned action and planned behavior and information–motivation–behavioral skills model, as well as the transtheoretical model.^10-22^ In this study, we examine how self-efficacy, a common construct among the afore-mentioned theories predicts safe-sex practices. Self-efficacy refers to self-confidence for behavior engagement in the presence of barriers militating against engaging in the desired behavior.^23,24^ This is influenced by factors such as mastery, vicarious experiences (modeling), verbal encouragement, and physiological states.^24^ Self-efficacy has shown some degree of association with physical activity behavior^23^ and smoking cessation behavior^25^ from previous research. However, its application with safe sex behavior is still a subject for ongoing research such as this study.

Self-efficacy for condom use refers to the confidence a college student has in his/her ability to engage in condom use in the presence of situation-specific barriers to condom use, such as the influence of alcohol, partner disapproval or the heat of the moment.^11^ Previous research has shown varying domains of safe-sex self-efficacy to be predictive of safe-sex practice.^8,11-13^ Brafford and Beck demonstrated some evidence of discriminant validity using a 28-item Condom Use Self-Efficacy Score (CUSES); they found significant differences in scale score between condom users and nonusers.^13^ However, Brien et al found a factor structure of condom use self-efficacy among three distinct groups of condom users in a college setting, with four factors identified including mechanics, partner’s disapproval, assertiveness and intoxicants.^12^ Collectively, this underscores the importance of future work on this topic assessing varying domains of safe-sex self-efficacy, which is less common in the literature. Within this context, mechanics-related self-efficacy refers to individuals’ confidence in their ability to properly wear a condom on themselves or partner; partner’s disapproval-related self-efficacy refers to the ability to suggest using a condom while bearing in mind the potential for rejection or misinterpretation; assertiveness-related self-efficacy refers to individuals’ confidence in their ability to openly dialogue condom use with their partners; and intoxicants-related self-efficacy refers to the self-confidence for condom use while under the influence of alcohol or other intoxicants, or in the heat of passion.

In our study, we assess the association of these four domains of self-efficacy on safe-sex practice in a Southern US college. We hypothesize that the examined domains of safe sex self-efficacy are associated with safe sex practices among college students. Such an investigation is important given the high rates of STIs in this population, and within this geographical region. Ultimately, delineating the link between domain-specific safe-sex self-efficacy and safe-sex practices may aid in the development, implementation, and evaluation of safe-sex interventions among this vulnerable population.

## Materials and Methods

### Study design & participants

We employed a cross-sectional study design using a Qualtrics online survey sent out via email to a random sample of college students at the author’s institution. This randomized sample of full-time students at both undergraduate and graduate levels in any of the five campuses affiliated with the author’s institution, was generated by the Office of Institutional Research, Effectiveness and Planning from a pool of all eligible students across these campuses. Consenting participants were asked to fill out a brief anonymous survey on variables such as demographics, safe sex practice, safe-sex self-efficacy, and self-esteem. In order to enable anonymity of the online survey, the survey was set up by the Office of Institutional Research, Effectiveness and Planning such that no identifying information was associated with the responses. Of the 266 participants who completed the survey, 157 were eligible (sexually active and of any sexual orientation exclusive of women who have sex with women) and had complete data for the variables assessed. Descriptive characteristics of these 157 participants are as displayed in Table 1. Further exclusion of individuals who reported no sexual activity in the past 30 days resulted in a sample size of 137. Similar sample sizes have been utilized in previous research on this topic.^8,14^ For example, Kanekar and Sharma^14^ demonstrated that among 150 African-American college students, self-efficacy towards safer sex was associated with safe sex practices.

### Demographic variables 

As similarly utilized elsewhere,^8,12^ participants were asked questions about the following demographic variables: age, gender, race/ethnicity, college level, locality (domestic or international student), cumulative grade point average (CGPA), alcohol consumption, present relationship status, if they had ever taken a sexuality class, if they had ever had sexual intercourse (those who had never had sexual intercourse were excluded from our analysis), age at first sexual intercourse, number of sexual partners in past 30 days and if they had ever been diagnosed with a sexually transmitted disease.

### Sexual intercourse definition 

Sexual intercourse was defined for the purpose of our study as: “vaginal or rectal penetration by human penis.”^8,15^

### Safe sex practice survey

The safe sex practice survey, as utilized elsewhere,^8,14^ includes 5 items assessing safe sex practice behavior. For example, participants were asked: “During the past 30 days, when having sexual intercourse, how often do you use condoms? (Never/Hardly ever/Sometimes/Almost always/Always/Not sexually active in past 30 days).” Excluding individuals not sexually active in past 30 days, scores range from 5–25, with higher scores representing a higher level of safe sex practice. These items have demonstrated some evidence of convergent validity by associating with constructs from the social cognitive theory.^8,14^ Cronbach α for the safe sex practice survey was 0.82 in our sample.

### Safe sex self-efficacy 

Safe sex self-efficacy scale was assessed using the Condom Use Self-Efficacy Scale (CUSES). The shortened version utilized includes 15 questions related to an individual’s confidence that they can use (or insist their partner uses) a condom in the presence of barriers related to condom use,^13^ with response options rated on a 5-point Likert scale ranging from strongly disagree (1) to strongly agree (5). This self-efficacy scale was developed based on 4 identified factors: *mechanics* (sample question - I feel confident in my ability to put a condom on myself or my partner); *partner’s disapproval* (sample question – If I were unsure of my partner’s feelings about using condoms, I would not suggest using one); *assertiveness* (sample question - I feel confident in my ability to discuss condom usage with any partner I might have); and *intoxicants* (sample question - I feel confident that I would remember to use a condom even if I were high). This CUSES adapted from a larger 28-item scale,^13^ has shown some evidence of reliability and validity - for example, Cronbach alphas for the four factors utilized range from 0.78 to 0.82.^12^ Cronbach alphas in our sample were 0.90 (mechanics), 0.89 (partner disapproval), 0.80 (assertiveness), 0.83 (intoxicants). With respect to validity, these items have been shown to be predictive of condom use among three distinct groups of condom users-ritualistic, sporadic and non-users.^12^ Factor analysis of the safe sex self-efficacy items identified four factors with factor loadings for mechanics’ items ranging from 0.70–0.91, partner disapproval 0.54–0.92, assertiveness 0.66–0.76, and intoxicants 0.69–0.74. This is similar to the factor structure identified elsewhere.^12^

### Self-esteem

Self-esteem was assessed using a 1-item measure as follows: “I have high self-esteem (rating from 1-not very true of me, to 7-very true of me)” Though shortened, this scale has shown convergent validity with the Rosenberg Self-Esteem Scale.^16^

### Statistical analyses

All statistical analyses were computed using Stata version 12 (Stata Corp College Station, TX). A multivariable logistic regression model was used to examine the association between safe sex self-efficacy (predictor variable; score range 15-75, higher scores represent higher reported condom-use self-efficacy), and safe sex practice (outcome variable dichotomized using the median split, with the median value being 19). Covariates in the regression model included all demographic variables described above, as well as self-esteem (score range 1-7, higher scores represent higher reported self-esteem). We also utilized similar separate models to examine the association between different domains of safe sex safe sex self-efficacy (mechanics, partner’s disapproval, assertiveness and intoxicants) and safe sex practice. Multiplicative interaction analyses were employed to examine the joint effects of safe sex self-efficacy and demographic variables on safe-sex practice. Statistical significance was set at α=0.05.

## Results

Among the participating 157 college students, the mean (95% CI) age was 22.77 years (21.82–23.73); mean age at first sexual intercourse was 17.18 years (16.75–17.62); the average number of sexual partners in the past 30 days was 1.10 (1.00–1.19). In the sample, 34.39% were male and 65.61% female; 80.25% were White while 19.75% comprised of other racial/ethnic groups; 12.74% were married, 52.22% reported being in a steady monogamous relationship, 12.10% in an unsure-monogamous/casual relationship and 22.93% were not in a relationship; 62.42% had never taken a sexuality class; and 8.92% reported a previous STI diagnosis by a clinician. 69% of respondents reported being monogamous over the past 30 days, 27% reported always using a condom, and among those who reported some condom use over the past 30 days, 61% reported always leaving space at the end (6% never left space).

Table 2 displays the logistic regression models examining the association between safe-sex self-efficacy (independent variable) and condom use. Model 1 represents the composite condom-use self-efficacy score and condom use (safe-sex practice). Models 2–5 represent the isolated association of each domain of condom use self-efficacy (mechanics, partner disapproval, assertiveness and intoxicants respectively) with condom use. For all analyses, with the exception of mechanics’ self-efficacy (Model 2) and assertiveness self-efficacy (Model 4), there was a significant association between condom use self-efficacy and safe-sex practices (*P*<0.05).

For every 1-unit increase in the composite condom use self-efficacy score, there was an 8% increase in the odds of being beyond the median safe-sex practice score (odds ratio [OR]: 1.08, 95% CI: 1.02-1.15). The mean variance inflation factor for this composite model was 1.33 (range 1.07–1.72), demonstrating no evidence of multicollinearity in this model. Among the analyzed individual domains of condom-use self-efficacy, self-efficacy for intoxicants use appeared to have the highest associated increase in odds of safe-sex behavior; for every 1-unit increase in intoxicants self-efficacy score, there was a 31% increase in the odds of being beyond the median safe-sex practice score (OR: 1.31, 95% CI: 1.08-1.58). With respect to partner disapproval, there was an associated 18% (OR: 1.18, 95% CI: 1.02-1.36) increase in odds of being beyond the median-split for condom use with every 1-unit increase in safe-sex self-efficacy scores. Neither condom use mechanics (OR: 1.08, 95% CI: 0.92-1.26) nor assertiveness self-efficacy (OR: 1.19, 95% CI: 0.91-1.54) were statistically associated with condom use self-efficacy. There was no multiplicative interaction effect between age and safe sex self-efficacy (OR: 1.01, 95% CI: 0.99-1.03), gender and safe sex self-efficacy (OR: 0.98, 95% CI: 0.87-1.10), safe sex self-efficacy and college level (OR: 1.02, 95% CI: 0.99-1.06), safe sex self-efficacy and race-ethnicity (OR: 1.01, 95% CI: 0.84-1.20), or safe sex self-efficacy and relationship status (OR: 1.02, 95% CI: 0.96-1.07).

## Discussion

The purpose of this brief report was to examine the association of safe-sex self-efficacy and safe-sex practice in a Southern US college. Given that the Southern region of the United States has about the highest burden in prevalence of STIs,^4-6^ as well as the highest incidence also reportedly seen in individuals of college age,^2,3^ it is pertinent that we identify predictors of safe-sex behavior in this particular demographic (Southern college-age students). Although safe-sex self-efficacy, which refers to the confidence an individual has in their ability to engage in safe-sex practices in the presence of barriers to this behavior,^13^ has been shown to associate with safe-sex practice among African-Americans^14^ and college-age students in a Northeastern University,^8^ it was important to examine this theoretical framework in this demographic on the basis of the aforementioned STI burden as well as the non-generalizability of earlier studies in other populations.^8,14^ Our findings show some consistency with previous research with respect to the association of composite safe-sex self-efficacy and safe-sex practice.^8,14^

While examining four domains of safe-sex self-efficacy, which is less common in the literature, we observed an apparently greater association between intoxicants self-efficacy and safe-sex practice. This may be informative as to the comparatively higher need for college-age students to be taught methods to enhance safe-sex practice in the presence of intoxicants. The college years are peculiar as the age for increased exploration and experimentation with sex and alcohol.^7^ Additionally, the Southern region of the United States has among the highest reported intensity of binge drinking in the country.^17^ Previous research suggests that alcohol use in this college setting occurs more during the weekends.^18^ Also notable in our institution is that in 2015, 32% of college students who consume alcohol reported having unprotected sexual intercourse while drinking alcohol within the past year.^19^ Learning skills to enhance safe-sex behavior in the presence of intoxicants may therefore be a good approach to include in working towards greater control of the STI burden in Southern US college students.

Additionally, we observed significant associations between self-efficacy for partner disapproval (an individual’s confidence in their ability to use a condom in the presence of partner disapproval) and safe-sex practice. There was no statistically significant association between the closely-related assertiveness self-efficacy scale and safe-sex practice in this study; however, poor assertiveness and low perceived personal control are thought to be predictors of risky sexual behavior.^20-22^ Hence, college students also need to build their self-assertiveness skills during this critical period of psychosocial development and our findings further emphasize the importance of good partner disapproval-related self-efficacy in relation to safe sexual behavior.

Although we observed no statistically significant association between self-efficacy for mechanics of condom use (confidence in ability to correctly use a condom) and safe-sex practice, worthy of note was that 62.42% of participating students reported never having taken a sexuality class. It would therefore be fallacious to make any assumptions about the ability to properly use condoms in this population (for instance, 33% of respondents reported never leaving space at the end of the condom) and this may be a pointer to the need for the integration of safe-sex related topics in relevant aspects of the college curriculum as well as during institutional health fairs. These findings buttress the importance of continuing safe-sex education at the college level. Although 69% of respondents reported being monogamous over the past 30 days, only 27% of individuals reported always using a condom. Continued open dialogue about sexual intercourse and safe-sex practices between these young adults and their tutors/professors who may serve as models, may enhance their confidence (and perhaps generate some automaticity) with discussion of the subject matter with potential sexual partners, especially when under the influence of alcohol or other intoxicants, or when the potential partner opposes the use of a condom. Hence, policy makers should consider the continuation of education on sexual behavior at the college level. Additionally, sex educators, in addition to improving the knowledge of their students on sexual behavior, should also aim to build their confidence that they can put learned concepts into practice, especially in precarious situations.

Limitations of our study include the cross-sectional methodology employed which provides limited information on the temporality of these associations. However, there is great plausibility for the observed association between safe-sex self-efficacy and safe-sex practices. The use of a one-item tool in assessing global self-esteem may be limited in ascertaining more psychodynamically complex constructs such as narcissism.^16^ Additionally, although our study examined data from five campuses associated with our institution, a larger sample size would have provided a more representative picture, hence there is limited generalizability of our findings to other Southern colleges or other areas across the country. Strengths of our study include the examination of composite as well as isolated domains of safe-sex self-efficacy on safe-sex practice. The interaction between safe sex self-efficacy and other psychological (such as affective)^26^ and interpersonal dimensions^27^ that may also influence sexual behavior may be worthy of future investigation.

## Conclusion

In conclusion, we observed a consistent association between safe-sex self-efficacy and safe-sex practices. This consistency with previous research is informative for development of targeted approaches to foster safe-sex behavior in US colleges. Future research may employ a longitudinal approach in order to examine temporal sequence of the examined associations.

## Ethical Approval

This study was approved by the ethics committee of the authors’ institution.

## Competing interests

We declare no conflicts of interest.

## Authors’ contributions

All authors were involved in the conceptualization of the study, revising the manuscript and interpreting the results. Author OA computed the analyses and drafted the first draft of the manuscript.

## Acknowledgments

No funding was used to prepare this manuscript.

**Table 1:** Demographic characteristics of the analyzed sample (N = 157)

**Characteristics**	**Sample mean (95% CI) or proportion (%)**
Age (years), range 18–58	22.77 (21.82-23.73)
Gender (%)	
Male	34.39
Female	65.61
Race/ethnicity (%)	
White	80.25
Other	19.75
College Level (%)	
Freshman	14.01
Sophomore	17.19
Junior	24.84
Senior	13.37
Super senior (5th year and above)	5.09
Graduate	25.48
Current CGPA (%)	
1-<3	21.65
3-3.6	36.31
3.6 and above	42.04
Alcohol consumption (drinks/week)	3.47 (3.15-3.79)
Current relationship status (%)	
Married	12.74
Steady monogamous	52.22
Casual/unsure monogamous	12.10
Not in a relationship currently	22.93
Ever taken a sexuality class (%)	
Yes	37.57
No	62.42
Age at first sexual intercourse (years), range 1-25	17.18 (16.75-17.62)
No. of sexual partners in past 30 days, range 0-5	1.10 (1.00-1.19)
Previous STI diagnosis by a clinician (%)	
Yes	8.92
No	91.08
CUSES, range 15-75	64.18 (62.83-65.54)
Self-esteem, range 1-7	5.24 (4.98-5.49)

Abbreviations: CGPA, cumulative grade point average; STI, sexually transmitted infection; CUSES, Condom Use Self-Efficacy Score.

**Table 2:** Logistic regression models examining the association between safe-sex self-efficacy (independent variable) and condom use^a^ (N = 137)

**Model**	**OR**	**95% CI**	**P value**
1. Condom Use Self-Efficacy Score, 1 unit increase	1.08	1.02-1.15	0.011
2. Mechanics Self-Efficacy, 1 unit increase	1.08	0.92-1.26	0.355
3. Disapproval Self-Efficacy, 1 unit increase	1.18	1.02-1.36	0.023
4. Assertiveness Self-Efficacy, 1 unit increase	1.19	0.91-1.54	0.200
5. Intoxicants Self-Efficacy, 1 unit increase	1.31	1.08-1.58	0.005

Abbreviation: OR, odds ratio. ^a^Condom use stratified by median split (median score = 19). Five separate models (Models 1, 2, 3, 4 and 5) were computed to examine the association between safe-sex self-efficacy and condom use (outcome variable for all 5 models). The predictor variable for Model 1 was the composite self-efficacy score; for Model 2, the mechanics self-efficacy score; for Model 3, the disapproval self-efficacy score; for Model 4, the assertiveness self-efficacy score; for Model 5, the intoxicants self-efficacy score. Covariates in each of the five regression models were age (years, continuous), gender (categorical), race-ethnicity (categorical), college level (categorical), CGPA (categorical), relationship status (categorical), alcohol use (continuous), age at first sexual intercourse (continuous), ever taken a sexuality class (categorical), ever diagnosed with STI (categorical), number of sexual partners in past 30 days (continuous), self-esteem (continuous).
